# Preferences for end-of-life care: A nominal group study of people with dementia and their family carers

**DOI:** 10.1177/0269216312464094

**Published:** 2013-05

**Authors:** Karen H Dening, Louise Jones, Elizabeth L Sampson

**Affiliations:** Dementia UK, London, UK; Marie Curie Palliative Care Research Unit, UCL Mental Health Sciences Unit, University College London, London, UK; Marie Curie Palliative Care Research Unit, UCL Mental Health Sciences Unit, University College London, London, UK; Marie Curie Palliative Care Research Unit, UCL Mental Health Sciences Unit, University College London, London, UK; Barnet, Enfield and Haringey Mental Health Trust, London, UK

**Keywords:** Advance care planning, dementia, end of life, nominal group technique, prospect theory, palliative care

## Abstract

**Background:**

The wishes and preferences of people with dementia should inform decisions on future care. However, such decisions are often left to family carers and may not reflect those the person with dementia would have made for themselves. We know little about what influences agreement between people with dementia and their carers.

**Aim:**

To explore whether people with dementia and their carers were able to generate and prioritise preferences for end-of-life care. We examined whether carers influenced the choices made by the persons with dementia.

**Design:**

Nominal group technique.

**Setting/participants:**

People with dementia (*n* = 6), carers (*n* = 5) and dyads of people with dementia and carers (*n* = 6) attending memory assessment services.

**Methods:**

Three modified nominal group technique groups were conducted in five stages: (1) silent generation of ideas, (2) discussion, (3) further generation of ideas, (4) discussion and themeing and (5) ranking. The discussions were recorded, transcribed and analysed for thematic content using NVIVO8.

**Results:**

Quality of care, family contact, dignity and respect were ranked as significant themes by all groups. The analysis of transcripts revealed three main themes: quality of care, independence and control and carer burden. People with dementia had difficulty considering their future selves. Carers wanted much control at the end of life, raising issues of assisted dying and euthanasia.

**Conclusions:**

Wishes and preferences of people with dementia and their family carers may differ. To ensure the wishes of people with dementia are respected, their views should be ascertained early in the disease before their ability to consider the future is compromised.

## Introduction

Advance care planning (ACP) is a ‘process of discussion that usually takes place in anticipation of a future deterioration of a person’s condition, between that person and a care worker’ usually from a health-care background.^1^ Developed largely in the United States, Australia and Canada, ACP is a process of communication that involves people in decisions about future care, making plans to ensure their preferences can be met when their mental capacity is lost.^2^

ACP is less developed in Europe and the United Kingdom, where historically its legal status has been unclear.^3,4^ In England and Wales, it is recognised that, under common law, a specific anticipatory statement (usually advance refusal of medical treatment) has legal status. The Mental Capacity Act 2005 ^5^ seeks to ensure that people without mental capacity are enabled to make their wishes and preferences for care and support known, so that these will be carried out.

Evidence for the effectiveness, feasibility or acceptability of ACP for people with dementia is limited.^6,7^ Some research has explored whether a ‘window of opportunity’ exists before capacity and cognition to undertake this process are lost.^8,9^ However, many people with early dementia may already be too cognitively impaired at the time of diagnosis to complete ACP themselves.^10^ Family carers’ involvement in medical decision-making increases as patient involvement declines.^11^ Family carers are expected to act as ‘proxies’, making difficult and emotionally demanding decisions^12^ and often suffering significant distress, in particular ‘anticipatory’ or ‘pre-death’ grief.^13^ Feelings of guilt and failure, together with insufficient information about the course of the disease, often leave family carers unprepared to make end-of-life decisions on behalf of their relative.^14,15^ It remains unknown whether the decisions that carers make accurately reflect those the person with dementia would themselves have made, or how carers are influenced by their own wishes.

This article describes the use of a modified nominal group (NG) technique to examine (1) how people with dementia define their wishes and preferences for their end-of-life care, (2) how family carers define preferences for their own end-of-life care and (3) whether the expression of the wishes and preferences of the person with dementia is facilitated or inhibited by the carer being present.

## Method

The NG technique^16^ is a structured evaluative methodology, developed to facilitate group or team decision-making. It has been used in health-care settings for those with impaired language, understanding and capacity.^17,18^

We recruited a purposive sample, representative of the local population, from the Memory Service in Barnet, Enfield and Haringey Mental Health National Health Service (NHS) Trust to participate in three NGs: (1) people with dementia, (2) carers of people with dementia and (3) dyads of people with dementia and their carer. Each participant attended one NG. Each NG lasted up to 90 min.^19^

### Inclusion criteria

The participants with dementia had a clinical diagnosis,^20^ a Mini-Mental State Examination (MMSE)^21^ score of >20^6^ and capacity to consent to participate.^22^ The carers were unpaid, not acting in a professional capacity and usually family carers who were ‘key decision makers’.

### Exclusion criteria

We excluded people with dementia without capacity to consent and those unable to communicate sufficiently in English as funding for interpreters was unavailable.

### Information and consent for participants

The capacity assessment of the person with dementia was made by both referring clinician and researcher, using guidance from the 2005 Mental Capacity Act.

### Ethical considerations

The study was approved by the Barnet, Enfield and Haringey Local Research Ethics Committee in 2009 (09/H0723/2).

### Conduct of the groups

To ensure consistency, the NGs were conducted according to a predetermined schedule, which included introductory text and a basic introduction to ACP.

#### Stage 1: generation of ideas (10 min)

The participants were asked to consider what their preferences for care would be if their health deteriorated and death approached and to write a word or short statement for each onto a ‘post it’ note. The number of ideas they could generate was not limited. This stage aims for silent generation of ideas,^16,23^ but remaining silent may not be conducive to a supportive environment for people with dementia, so we offered reminders of the purpose of the group.

#### Stage 2: discussion (15 min)

This involved a structured and time-limited discussion of all ideas generated.^23^ This was to clarify ideas, explore the underlying rationale and add further items that emerged through discussion, ensuring that each participant felt that their contributions were valued. Ideas were placed on a central board in full view of all participants.

#### Stage 3: further generation of ideas (10 min)

The participants were asked to consider any additional ideas arising after hearing those of others.

#### Stage 4: discussion and generations of themes (10 min)

All contributions were discussed to generate common themes. Finally, each group formulated statements to reflect the themes. This group activity ensured face validity of themes.

#### Stage 5: individual ranking (10 min)

**Figure 1.**
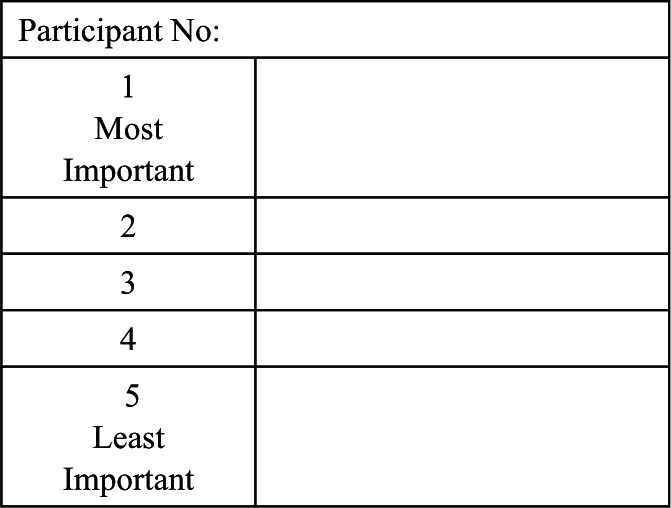
Ranking sheet.

Rather than voting collectively, ranking was undertaken individually to allow all members to determine their own priorities. This enabled people with dementia to express their preferred choices independently of other group members. The participants ranked their five most important items (high = 1, low = 5; Figure 1).

At the close of each NG, participants were given a booklet on ACP^24^ with the option of contacting the Admiral Nursing service if they wished to develop an advance care plan. All NGs were audiotaped and later transcribed verbatim.

## Data analysis

Data on the baseline demographic characteristics of participants were recorded. The qualitative data were collected from three NGs held in the memory clinic on different days from October 2009 to January 2010. The researcher (K.H.D) was assisted by a specialist dementia (Admiral) nurse to support individuals who required help during or after the group.

We took two approaches to ensure face and content validity of the emerging themes:

1. Collation of themes and scoring of the individual ranked items.
2. Qualitative content analysis of discussion transcripts.

### Scoring of individuals’ ranking of themes

The researcher assigned a score to each of the five highest individually ranked items to identify summative ranked priorities for each group. The scoring system used allowed overall priorities to be identified (highest ranking = 10; lowest ranking item = 2). A group score for each item raised in each group was derived by summing the individual scores for each, which then provided a ranking of items from each of the three groups. Finally, all group scores were collated to give an overall priority of items from all three groups.

### Qualitative content analysis

Emerging patterns were identified, coded and categorised from the data.^25^ The data were divided and organised, within NVIVO8,^26^ supported by manual coding and themeing independently and then collectively by the researcher (K.H.D) and supervisor (E.S) to ensure reliability and validity. The data tree and themes were then agreed upon by K.H.D and E.S.

The results are presented as graphs to reflect ranked and scored items supported by analysis of the discussions held during the NG processes.

## Results

### Participant characteristics

Twenty-six people agreed to participate, of whom 17 attended: nine people with dementia and eight carers. Decisions for non-attendance were made by carers, either for themselves (NG 1) or on behalf of the dyad (NG 3). All invited participants of NG 2 attended. Mean age of people with dementia was 83.3 years and carers 69.2 years. Mean MMSE of people with dementia was 24.2. Most participants (*n* = 11) were educated beyond school leaving age (see Table 1).

**Table 1.** Characteristics of participants (*N* = 17).

	PWD	Carers
Age (overall)	83.3 years (*n* = 9)	69.2 years (*n* = 8)
Dementia NG 2	83.3 years (*n* = 6)	
Carer NG 1		66.8 years (*n* = 5)
Dyad NG 3	82.3 years (*n* = 3)	73.3 years (*n* = 3)
MMSE (overall)	24.2	
Dementia NG 2	24.1	
Dyad NG 3	24.5	
Diagnosis		
F00.1 (Alzheimer’s late onset)	4	
F00.2 (atypical or mixed type Alzheimer’s)	4	
F00.9 (Alzheimer’s of unspecified type)	1	
Gender		
Male	3	3
Female	6	5
Ethnicity		
White British	5	5
White European	1	2
White American	1	1
Black Caribbean	1	–
Asian British	1	–
Previous education		
Degree or above	2	6
College	3	–
Left school with no qualifications	4	2
Living situation of PWD		
Alone	4	
Spouse	3	
Child	1	
Sibling	1	
Relationship to PWD		
Spouse		5
Son/daughter		2
Sibling		1

PWD: people with dementia.

### Process

We found that people with dementia required reminders and support from the groups’ co-facilitator (Admiral Nurse) at each stage of the process. People with dementia in NG 2 required most help. In NGs 1 and 3, family carers tended to intervene if the person with dementia required support in all but the ranking stage (stage 5).

#### NG 1: carers of people with dementia

**Figure 2.**
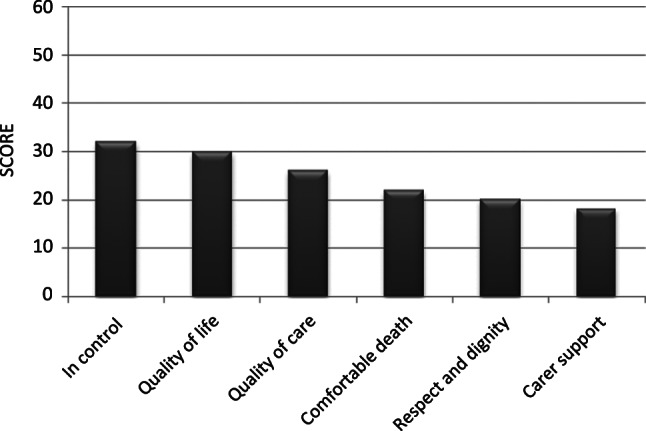
Ranking of items: nominal group 1 (carers of people with dementia).

In NG 1 (*n* = 5), the ranking of items in order of priority (Figure 2) was as follows: to be in control, to have a good quality of life, to have good quality care, to have a comfortable death, to be treated with respect and dignity and to have carer support.

#### NG 2: people with dementia

**Figure 3.**
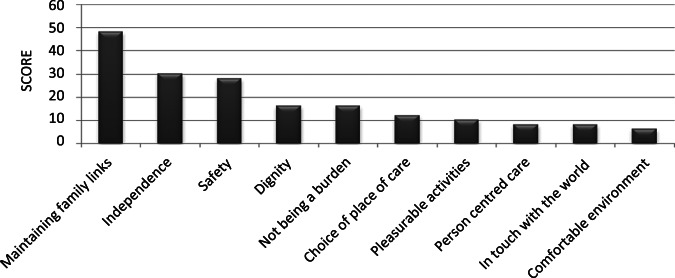
Ranking of items: nominal group 2 (people with dementia).

In NG 2 (*n* = 6), the ranking of items in the order of priority (Figure 3) was as follows: to maintain family links, to maintain independence, to feel safe, not to be a burden, to be treated with respect and dignity, to have a choice in the place of care, pleasurable activities, person-centred care, to be in touch with the world and to have a comfortable environment.

#### NG 3: dyads of carers and people with dementia

**Figure 4.**
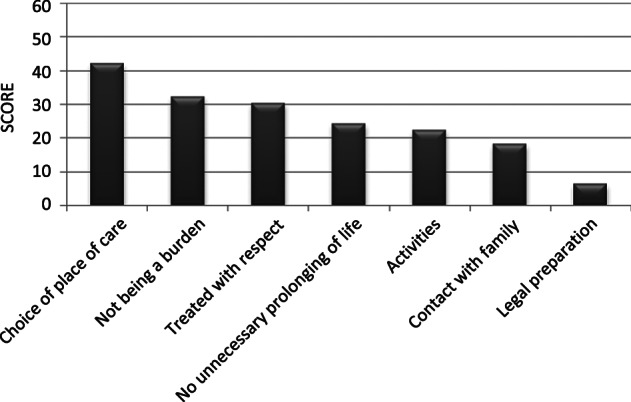
Ranking of items: nominal group 3 (dyads: people with dementia and their main carer).

In NG 3 (*n* = 3 dyads), the ranking of items in order of priority (Figure 4) was as follows: choice of place of care, not to be a burden, to be treated with respect and dignity, no unnecessary prolonging of life, to be active, to maintain contact with family and to make legal preparation.

**Figure 5.**
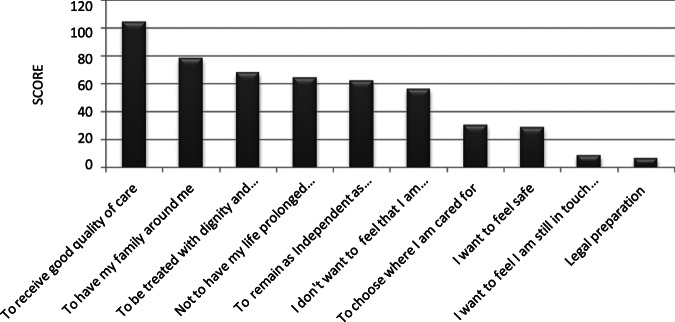
Final ranking of all statements.

Overall priorities were calculated from scores of individually prioritised items (Figure 5). The three highest ranked items of the combined scores from all three NGs were the wish to receive good quality care, to have one’s family close by and to be treated with dignity and respect at the end of life.

Of the 17 participants, two carers and one person with dementia contacted the Admiral Nursing Service within a month of their NG to enquire further into ACP.

### Emergent themes

The themes arising from individual ranking and content analysis of discussions did not differ significantly, which ensured content validity.

### Good quality care

The most prominent theme for all participants was the wish to receive good quality care at the end of life, with carers hoping for continuing control over this. In describing good quality care, people with dementia talked of their lives very much in the ‘here and now’, the elements being activities they currently enjoyed and valued. They seemed unable to consider their *future self* or that at some point valued activities would alter or cease altogether:

… well I go back and forth [to Ireland] … I will continue to do that … Person with dementia 05 (NG 2)

Carers talked extensively about their perceptions of poor quality care, based upon recent media coverage and reflections of personal experiences of caring for a person with dementia, and framed this as care that they *would not* want for themselves. Several spoke of care that was ‘desirable’:

… non institutionalised care … carer support to stay at home … it should be with one person coming in … things that appear not to be important and unrelated to health but actually take a much higher place. Carer 04 (NG 1)

People with dementia made some reference to quality care:

… look after me with care … don’t treat me like a vegetable … like a mad person. Person with dementia 01 (NG 3)

All NGs mentioned dignity and respect as integral to good quality care and future wishes, but participants found these difficult concepts to define. The carers felt that poor care arose from an underlying lack of respect for people with dementia, which robbed them of dignity.

### Independence and control

The participants with dementia saw ‘independence’ as a broad and intangible aspect of their future, making assumptions that they would retain independence.

The carers (NG 1) considered a future time when they themselves might lack decisional capacity. There was a general fear and uncertainty with a lack of trust in medical decision-making:

… being sure that treatment is in my best interests … It means that you have got to trust in people who make the decision … Carer 01 (NG 1)

In the dyad group (NG 3), carers tended to speak on behalf of the person with dementia, thus influencing the collective view. The people with dementia found it difficult to consider preferences and wishes about their end of life, with little sense of the potential value of ACP or how expressing preferences and wishes *now* could influence care later.

… that’s a nice place to die … home … Person with dementia 02 (NG 3)

The carers felt it was difficult to plan ahead and anticipate what may or may not happen:

… you don’t know what changes will happen, when it will happen … that’s why it [ACP] is very difficult to define. Carer 05 (NG 1)

The carers felt that medical decision-making and the use of end-of-life care pathways could invalidate their ACPs:

… you are put on the short count to death row [End of Life Care Pathways] … I think a lot of elderly people are put on that path because it happens to be convenient … just because they are old basically, the plug is pulled … that decision can sometimes be made too early. Carer 03 (NG 1)

The carers expressed scepticism about whether an ACP would allow them to retain control. They thought that ACP may be a process with no firm outcomes open to (mis)interpretation by professionals:

… consolidates my slight fear about this sort of advanced care planning that it takes away [control] from individuals even though it’s prepared by an individual; you have to tick certain boxes. Carer 04 (NG 1)

Several carers (NG 1 and NG 3) felt the only way to ensure that control was retained was to take matters into their own hands through assisted dying and euthanasia. Once the topic had been raised, a growing confidence developed in NG 1 and many felt similarly, to the extent that one member used the term ‘suicide’. While acknowledging that euthanasia is not legal in the United Kingdom, NG 1 reached a consensus that you cannot discuss ACP without it.

… it is interesting for people to know in the back of their mind that even if it’s a subject we cannot go [not legal in the UK] that actually it looks as though quite a few of us were feeling that. Carer 02 (NG 1)

It was highlighted that if end-of-life care was better individuals would not need to contemplate euthanasia:

… I feel … that if you know and end of life sort of thing came up as sort of satisfying more people … possibly going down the suicide route would evaporate you know. Carer 03 (NG 1)

Whereas people with dementia in NG 3 felt that irrespective of the quality of care they would not want to continue living:

… change, feeding, some people [ … ] … is not right … if I am unwell and not enjoying my life and a vegetable [ … ] I would like to … I would be better off dead. Person with dementia 03 (NG 3)

… when I … am that bad … I would rather die … Person with dementia 01 (NG 3)

Summarising their views on ACP, NG 1 felt that carers’ needs should also be taken into account:

… it’s having support to whatever you want to do at the end, in the most comfortable way not only for you but also for your carers. Carer 01 (NG 1)

### Perceptions of burden and caring

Having continued contact with family, friends and loved ones in the future was highly valued by all groups. The people with dementia and carers discussed the nature of caring and not wanting to become a burden to their families. However, whereas the burden discussed by carers was subjective and based upon their current experiences, people with dementia had no perception of the sense of burden they generated on their carers and talked about burden as something that *may* occur in the future with little perception of the current situation:

[Burden] … only **if** I were totally dependent upon them. Person with dementia 01 (NG 2)

… well that what you get [to be a burden] … not there now … Person with dementia 02 (NG 2)

The people with dementia spoke positively about their families and saw the value of continued family contact. However, the carers in NG 3 often spoke over the person with dementia to point out that they did not want their children to find themselves in a similar position:

I don’t want to leave my son with things like that [making decisions and providing intimate care]. Carer 01 (NG 3)

Spousal carers appeared more accepting of their caring role, whereas siblings or children talked of the overwhelming difficulties of caring. One carer experienced such stress that should she also be affected by dementia, she had told her children that she wanted to go into a care home. She did not want her relationship with her children to be damaged by burden or responsibility:

… being a carer is difficult … it leaves some nasty memories … Carer 02 (NG 3)

The carers challenged ‘the system’ arguing that if health and social care were effective in supporting people with dementia and their carers, ‘burden’ would not be an issue.

## Discussion

Our main findings are that people with dementia find considering their preferences and wishes for end-of-life care challenging; the carers’ own preferences are influenced by current experiences of caring and in dyad groups, carers’ views tended to override those of people with dementia.

Both people with dementia and carers had difficulty with some concepts, for example, dignity and respect; terms often used liberally by professionals in ACP discussions. The people with dementia tended to think in a concrete way about future scenarios, and as in previous research, we found that they often frame their views solely in their present context.^27^ Thus, in practice, even people with early dementia may have difficulty in participating fully in ACP as they have to imagine their ‘future self’. Fazel et al.^8^ and Gregory et al.^28^ reported that MMSE scores of between 18 and 20 were required to make an ACP. However, our participants had a mean MMSE of 24.2 (range = 20–29) and most experienced difficulty with the concept of ACP, despite being educated to a higher level.

The carers’ own preferences were articulated within the context of their caring experience,^29^ often one that was negative and influenced by the nature and quality of the relationship with the person with dementia. Carers reflected on what their own future might hold based upon their perception of what it was like for their relative to *have* dementia, in a care system currently under much scrutiny and criticism^30^ and carer support that was inadequate.

While carers acknowledged some situations that may require specific decisions (e.g. care home admission, tube feeding, resuscitation), they felt such decisions would be made by health-care professionals irrespective of ACP and were beyond their own influence. Thus, the carers felt that ACP might not be sufficient to authorise them to act when the decisional capacity of the person with dementia was lost. Carers wished for autonomy for their own care if debilitating illness ensued, expressing a possible wish for assisted dying or euthanasia.

Despite evidence that ACP can contribute to the quality of remaining years in life-limiting conditions,^31^ guide family members^3^ and take the ‘burden’ out of making end-of-life decisions,^32^ it may still be limited in addressing future issues, either because of a desire to live in the present or because the prognosis is unclear.^33,34^ Apart from this, our work suggests that impaired cognitive function may bring additional problems, as people with dementia find it difficult to conceive their future self and possible burdens that their illness places on those around them. Also, little is known about issues of co-morbidity and ACP when dementia is one of those co-morbid conditions.

Making treatment decisions for older people is difficult when they lose the capacity to tell us what they want: a person needs to feel trust in a family carer’s ability to make such decisions,^35^ and they need to rely on their family members to indicate what their wishes and preferences might have been.^36^ Some studies have explored levels of agreement between people with long-term illness and their family carers and indicate varying levels of concordance.^37^ In a qualitative study of dyads involving heart failure, Retrum et al.^38^ found that lack of agreement could impair the ACP process.

## Strengths and limitations

Use of NGs allowed each participant an equal opportunity to contribute, supporting and valuing individual views. As each participant took part in one NG, we could not explore directly whether individuals with dementia behaved differently if their own carers were present.

Although our sample size was small and restricted to one locality, it represented a range of ethnicities, types of carers, living situations and levels of education (Table 1). Although not necessarily generalisable, our data were obtained directly from people with dementia and their carers. Although discussions did not cause overt distress, interaction in a group setting was limited and a ‘one-to-one’ approach might be more supportive. Carers tended to prioritise their own opinions, so we should be cautious if families speak for their older relatives, for example, if English is not their first language. However, without funding for interpreters, we were unable to explore this further.

## Conclusion

We have shown that the underlying wishes and preferences of people with dementia and their family carers may differ. The carers’ own wishes and preferences were shaped by their perceptions and experiences of dementia. While many carers believe that they are making decisions in accordance with the wishes of their family member, they may be making choices that are bound up with their own experiences and not concordant with those of the person with dementia had they retained capacity. One-to-one discussions between clinicians and people with dementia as early as possible to explore preferences, although limited in their range and depth, may be of most value in planning future care.
